# Extensive Subcutaneous Emphysema as a Presentation of Ischemic Colitis

**DOI:** 10.1155/2015/587508

**Published:** 2015-11-17

**Authors:** Ana Franky Carvalho, Claudio Branco, Pedro Leão, Conceição Antunes

**Affiliations:** ^1^Department of General Surgery, Hospital of Braga, 4710-243 Braga, Portugal; ^2^Life and Health Sciences Research Institute (ICVS), School of Health Sciences, University of Minho, 4710-057 Braga, Portugal; ^3^ICVS/3B's, PT Government Associate Laboratory, Guimarães, 4710-057 Braga, Portugal

## Abstract

*Introduction*. Subcutaneous emphysema is usually benign and self-limited; however, it may be associated with a life-threating situation.* Case Report*. An elderly woman with progressive malaise with extensive subcutaneous emphysema (cervical to abdominal wall) was observed at the emergency department. Colonic perforation was diagnosed and the patient underwent surgery. Intraoperatively, necrosis and perforation of the sigmoid colon into the retroperitoneum were found and a Hartmann procedure was performed.* Conclusion*. Cervical and thoracic subcutaneous emphysema may be the first sign of intra-abdominal lesion.

## 1. Introduction

Subcutaneous emphysema is characterized by swelling of the tissues due to accumulation and spreading of air along tissue planes. More often it suggests a communication between the subcutaneous tissue and an organ containing air (such as airway or viscera). However, it can be due to infection of subcutaneous tissue. Although it is usually benign and self-limited, it may be associated with a life-threating situation. The presence of thoracic or cervical subcutaneous emphysema may suggest damage to thoracic structures, while the presence of abdominal subcutaneous emphysema suggests damage of intra-abdominal viscera.

## 2. Case Presentation

**Figure 1:**
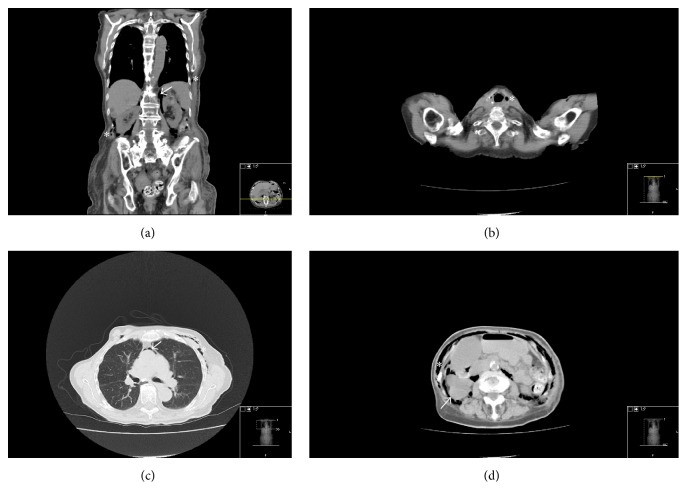
(a) Thoracic and abdominal (asterisk) subcutaneous emphysema and retroperitoneal gas (arrow). (b) Cervical subcutaneous emphysema (asterisk). (c) Thoracic subcutaneous emphysema (asterisk) and pneumomediastinum (arrow). (d) Abdominal subcutaneous emphysema (asterisk) and retroperitoneal gas (arrow).

A 93-year-old woman presented to the emergency department with malaise, progressive asthenia, and anorexia for 2 weeks and abdominal pain for 2 days. At physical examination, extended thoracic subcutaneous emphysema as well as cervical and abdominal subcutaneous emphysema was noted. The patient showed mild tenderness in the lower left abdominal quadrant. Computed tomography revealed subcutaneous emphysema in cervical, thoracic, and abdominal segments (*∗* in Figures 1(a)–1(d)). Moreover, gas in the retroperitoneum (arrows in Figures 1(a) and 1(d)) and pneumomediastinum (arrow in Figure 1(c)) was also visible. Images suggested a perforation of the sigmoid colon. An exploratory laparotomy was performed. Intraoperatively, multiple perforations of the sigmoid colon were found with fistulization to the retroperitoneum and anterior lateral abdominal wall with no visible colon diverticula. These perforations were probably related to ischemic events, as ischemic colitis, leading to necrosis of the colon. Resection of the sigmoid colon and an end colostomy were performed. Postoperatively, the patient showed good response to the treatment, with regression of the subcutaneous emphysema, and was discharged 16 days later.

## 3. Discussion

Supraclavicular subcutaneous emphysema is typically associated with a perforated gastric or duodenal ulcer, while cervical subcutaneous emphysema is usually associated with rupture of a thoracic organ or cervical esophagus or trachea. Subcutaneous emphysema is a rare but known manifestation of intra-abdominal pathology. There are several reports of abdominal and thoracic subcutaneous emphysema following iatrogenic perforation of the colon (by colonoscopy) [1, 2] or due to perforated diverticulitis [3, 4]. This patient had no history of recent colonoscopy or known diverticular disease. Ischemic colitis presents more often with abdominal pain and hematochezia. In this case, the combination of unspecific symptoms and extensive subcutaneous emphysema led to further investigation. Ischemic colitis originated necrosis and perforation of the sigmoid colon with fistulization to the retroperitoneum with little intra-abdominal spilling. The retroperitoneal gas, due to fistulization of the sigmoid colon to the left paracolic recess, ascended above the diaphragm through the diaphragmatic hiatus leading to the development of pneumomediastinum and thoracic and cervical subcutaneous emphysema. Cervical subcutaneous emphysema and pneumomediastinum may be the first manifestation of an occult abdominal perforation.
